# Immediate and transgenerational effects of thymol supplementation, inactivated Salmonella and chronic heat stress on representative immune variables of Japanese quail

**DOI:** 10.1038/s41598-020-74547-2

**Published:** 2020-10-23

**Authors:** E. A. Videla, O. Giayetto, M. E. Fernández, P. A. Chacana, R. H. Marín, F. N. Nazar

**Affiliations:** 1 Instituto de Ciencia y Tecnología de Los Alimentos (ICTA), Facultad de Ciencias Exactas, Físicas y Naturales, Universidad Nacional de Córdoba (UNC), X5000JJC Córdoba, Argentina; 2 Instituto de Investigaciones Biológicas y Tecnológicas (IIByT, CONICET-UNC), Consejo Nacional de Investigaciones Científicas y Técnicas (CONICET), X5000JJC Córdoba, Argentina; 3 Instituto de Patobiología, Instituto Nacional de Tecnología Agropecuaria (INTA), C1033AAE Buenos Aires, Argentina; 4 Present Address: School of Biology, Sir Harold Mitchell Building, University of St Andrews, St Andrews, Fife, KY16 9TH UK; 5 Present Address: Department of Animal Production, NEIKER-Basque Institute for Agricultural Research and Development, Vitoria-Gasteiz, Spain

**Keywords:** Animal physiology, Immunology

## Abstract

Environmental challenges are integrated in the inmunoneuroendocrine interplay, impacting the immune system of the challenged individuals, and potentially implying transgenerational effects on their offspring. This study addressed whether dietary supplementation with thymol can modulate the immune response of adult Japanese quail when simultaneously exposed to an inoculum of inactivated *Salmonella* Enteritidis and a chronic heat stress (CHS). We also evaluated whether the experienced situations by adults can affect the immune response of their undisturbed offspring. In the parental generation, supplemented quail exposed to CHS had a higher inflammatory response and similar values of the heterophil/lymphocyte (H/L) ratio than those that were not supplemented. In their offspring, those chicks whose parents were exposed to CHS showed higher inflammatory response and lower antibody production. Regarding the H/L ratio, chicks whose parents were supplemented showed lower H/L ratio values. Dietary supplementation with thymol partially and positively modulated the inflammatory response and avoided H/L ratio alteration in the parental generation exposed to high environmental temperatures, suggesting these adults were better at dealing with the challenge. The lower H/L ratio values in the offspring suggests that chicks are more capable to deal with potential stressful situations associated with conventional breeding conditions.

## Introduction

In vertebrates, many environmental challenges are physiologically integrated in the inmunoneuroendocrine (INE) interplay^1,2^. Once this interplay is activated, mediators (such as hormones or cytokines) are released in order to cope with these challenges. In avian species, elevated temperatures and the exposition to potentially harmful microorganisms are considered environmental challenges also integrated in the afore mentioned interplay, having major consequences on one of its components, the immune system^3–5^. These environmental challenges differentially affect both sexes, being males more susceptible than females^6,7^. However, the resulting physiological responses always demand resources aimed to reestablish the homeostatic conditions^8,9^ with consequences on the birds’ health status and welfare^3,10,11^. Providing the physiological possibilities of birds are limited, these consequences on the organism might be dependent on the differential allocation of resources, prioritizing efforts towards survival or reproduction, but having negative impacts on other systems^9,12,13^ leading to the manifestation of physiological trade-offs.

During summer months, especially in tropical and subtropical countries, recorded temperatures are above optima during daylight. In many cases, this scenario is sustained for several consecutive days^14,15^ leading to a phenomena described as chronic heat stress (CHS). The exposure to high environmental temperatures has a major impact on the physiology of poultry species, diminishing the growth rate and body weight gain, being associated with the reduced feed intake experienced by the birds under heat stress conditions^14,16^. On the other hand, the encounter of poultry species with an immune challenge, such as *Salmonella* ser. Enteritidis or *Escherichia coli*, had also effects on body weight and the feed conversion^17,18^. Additionally, potentially harmful microorganisms (i.e.: *Salmonella*) have been reported to continuously challenge birds’ immune system^19–21^. Previous reports inform that repeated episodes of elevated environmental temperature and high levels of pathogens challenge have detrimental effects on different components of the immune system. For example, alterations in white cells structure^22^, intestinal morphology and its microbiota^23^, increments in the heterophil to lymphocyte ratio (H/L), reductions in inflammatory responses^11,24^, macrophages activity^25^ and antibody mediated responses^22,25,26^ have been reported for poultry species.

Nutrition is also considered a potential environmental immune modulator. Different degrees of food deprivation increase corticosterone concentrations, cytokines related to pro-inflammatory processes and the H/L ratio^27,28^. In contrast, dietary supplements such as probiotics, prebiotics, or phytochemicals are able to positively modulate the immune system. In our particular study, we used thymol, one of the main components of the oregano´s essential oil. This component has been included in the diet of poultry species as a dietary supplement, improving the body weight gain, the oxidative stability properties of fatty acids, reducing fear-related behaviors but having no significant effects on the locomotion activity of birds^29–32^. Considering the immune system, thymol has been reported to increase the inflammatory response to phytohaemaglutinin-p (PHA-P)^33^, and together with its isomer, carvacrol, increase antibody titers against sheep red blood cells (SRBC) and decrease H/L ratio^34^. These results could be related to the many bioactive properties described for this particular phytochemical, such as immune-stimulator, antimicrobial, antifungal, and also interactions with neurotransmitter receptors^35–37^.

The environmental challenges have immediate consequences on birds that are being exposed to them, but, could also have transgenerational effects on their offspring^38,39^. Females transmit to their offspring several maternal components, such as hormonal, immune or even dietary components (e.g. carotenoids or thymol). These components would prepare the future offspring to develop in a similar environment where the parental generation was bred, according to the environmental matching hypothesis^1,2,32,40–44^. In avian species, females deposit all maternal components into the egg, gathering all the physiological information resulting from daily challenges^1^. The impact on the immune characteristics of the eggs seems to be related to the maternal component transferred. For example, the supplementation of grey partridges with carotenoids increased the concentration of lysozyme (an antimicrobial enzyme) in the egg albumen^40^. On another case, the exposure of laying hens to an immune challenge with lipopolysaccharides of *Salmonella* increased the antibacterial activity of the egg albumen against *Staphylococcus aureus*^45^. Lastly, hormones in eggs, such as testosterone, has been reported to affect the immunoglobulin concentration present in the albumen^43^.

Within the described complex scenario, this study addressed whether feed supplementation with thymol can positively modulate the immune response of adult Japanese quail when exposed to environmental challenges. Moreover, it also evaluates whether the experienced situations in the parental generation can affect immune representative variables on their eggs and offspring. Specifically, we evaluated the effects of inoculation with an inactivated *Salmonella* suspension and a CHS exposure on the inflammatory response to PHA-P, the production of antibodies against SRBC, and the H/L ratio in adult Japanese quail. Thereafter, same immune related variables were evaluated in undisturbed juvenile individuals, and antimicrobial and agglutinant activities were determined on the eggs.

## Results

### Parental generation

**Figure 1:**
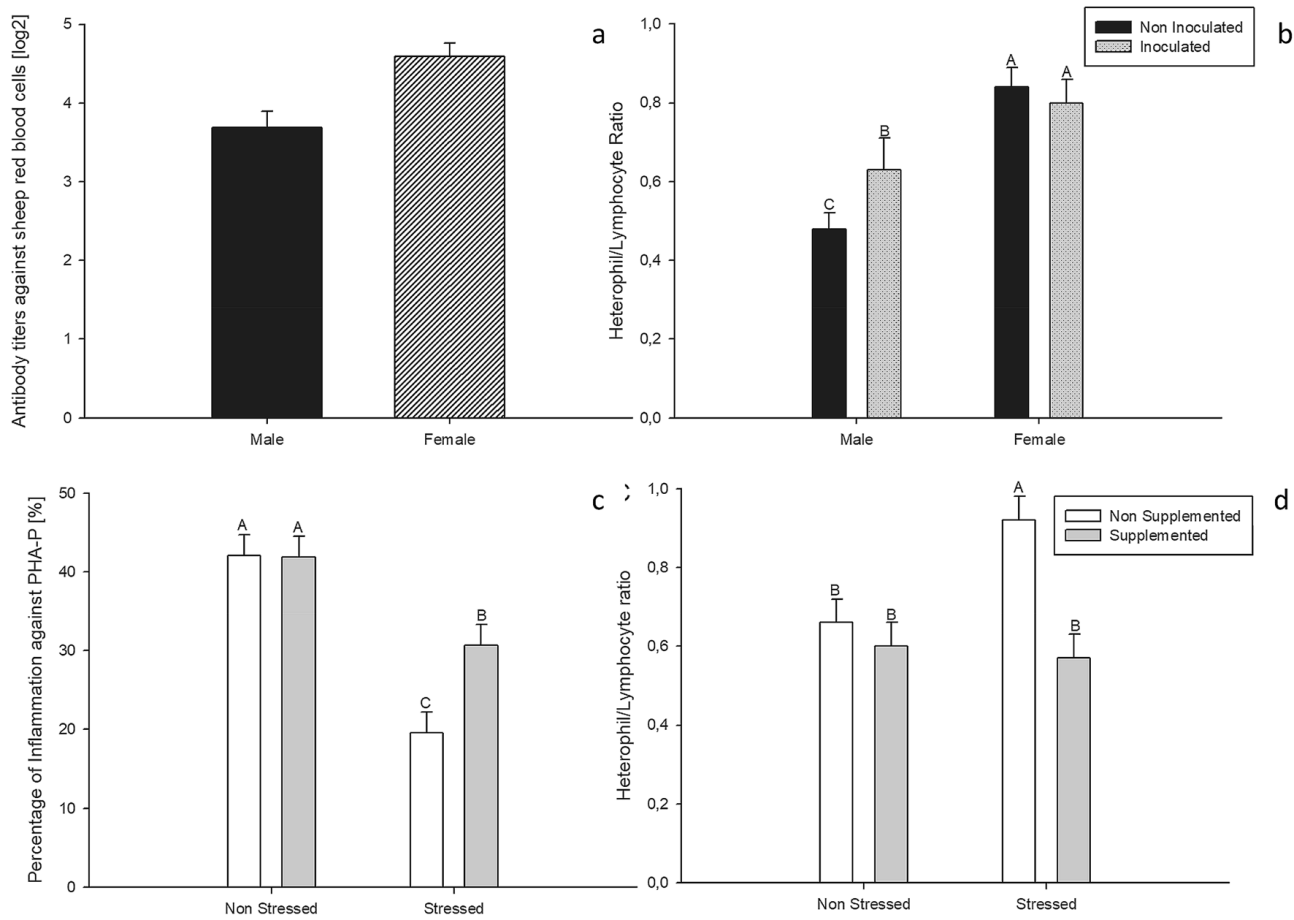
Effects of sex on the antibody titers against sheep red blood cells (**a**), sex and the inactivated *Salmonella* Enteritidis inoculum on the heterophil/lymphocyte ratio (**b**), thymol dietary supplementation and the environmental challenges on percentage of inflammation against PHA-P (**c**) and the heterophil/lymphocyte ratio (**d**) of 83 pair male–female adult Japanese quail. Dietary supplementation started at 103 days of age and lasted until the end of the study (130 days of age). Quail received a thymol dose equivalent to 80 mg/quail per day. At 115 days of age, quail were inoculated with Salmonella Enteritidis (SE) by an intramuscular injection. Chronic heat stress was achieved increasing room temperature from 24 to 34 °C during 9 consecutive days (121–129 days of age). Considering that no main effects of SE inoculation were detected, both SE and non-SE animals were pooled within the figures (**c**) and (**d**). Bars represent the mean value and lines represent standard error. ^A,B^Different letters represent statistical differences (P < 0.05) between groups.

The analysis of the titers against to SRBC showed no treatment effects nor interactions between them. Only a main effect of sex (F = 13.35, P < 0.01, Fig. 1a) was detected with females showing higher titers than males.

For the inflammatory response against PHA-P (Fig. 1c), the analysis showed a main interaction between the effects of dietary supplementation and CHS (F = 4.73; P = 0.03). Post-hoc analyses showed that quail exposed to CHS protocol had a lower inflammatory response to PHA-P. Interestingly, quail that were supplemented and exposed to CHS protocol had a higher inflammatory response than those that were not supplemented.

The analysis of the H/L ratio showed a main interaction between dietary supplementation and CHS (F = 5.14; P = 0.02, Fig. 1d). The post-hoc analysis showed that the highest H/L ratio was found in the group of quail that were not supplemented with thymol and exposed to CHS. Birds supplemented with thymol and exposed to the CHS protocol showed similar values than their non-stressed counterparts. A sex and inoculum interaction was also evidenced (F = 3.86; P = 0.05, Fig. 1b). Males inoculated with *Salmonella* Enteritidis showed higher H/L ratios than their non-inoculated counterparts. Females showed the highest H/L ratios regardless of whether they received the inoculum or not.

### Eggs

The number of eggs collected during the days number five, six, seven, eight and nine of the CHS protocol and the sampling day showed no differences between treatments (F < 2.04, P-values > 0.15).

**Figure 2:**
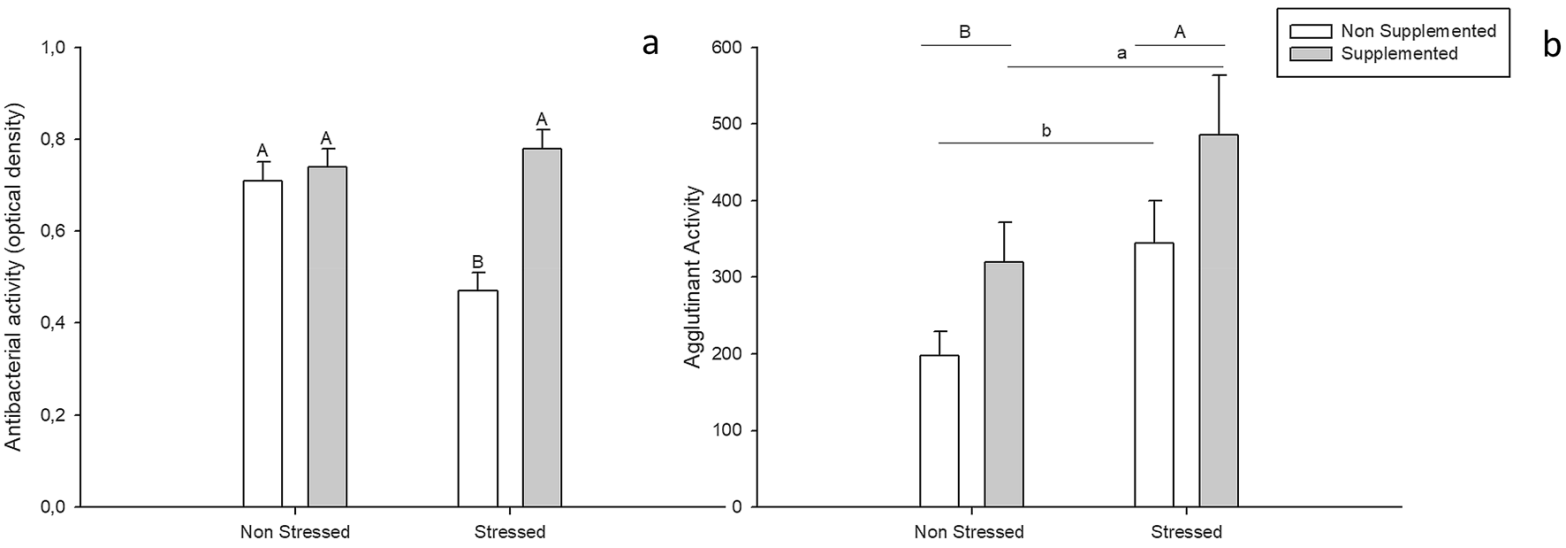
Effects of thymol dietary supplementation and the environmental challenges on the antimicrobial activity (**a**) and the agglutinant activity (**b**) in quail eggs. Dietary supplementation started at 103 days of age and lasted until the end of the study (130 days of age). Quail received a thymol dose equivalent to 80 mg/quail per day. At 115 days of age, quail were inoculated with Salmonella Enteritidis by intramuscular injection. Chronic heat stress was achieved increasing room temperature from 24 to 34 °C during 9 consecutive days (121–129 days of age). A total of 40 eggs (5 per treatment) collected on the 9th day of chronic heat stress were analysed. Bars represent the mean value and lines represent standard error. ^A–B/a–b^Different letters represent statistical differences (P < 0.05) between groups.

For the antibacterial activity (Fig. 2a), the analysis showed a main interaction between dietary supplementation and CHS (F = 9.92; P = 0.003). The post-hoc analysis showed that the eggs laid by females that were non-supplemented but exposed to CHS showed higher antibacterial activity than the eggs from the other 3 groups.

Regarding immunogenicity against *Salmonella* (Fig. 2b), the analysis showed main effects of dietary supplementation (F = 6.59; P = 0.001) and stress treatment (F = 9.17; P = 0.004), with no interaction between them. The eggs laid by females that were supplemented with thymol showed higher titers against *Salmonella* than their non-supplemented counterparts. The eggs from females exposed to CHS showed higher agglutination titers than the eggs from females reared under thermoneutral conditions.

### Offspring

**Figure 3:**
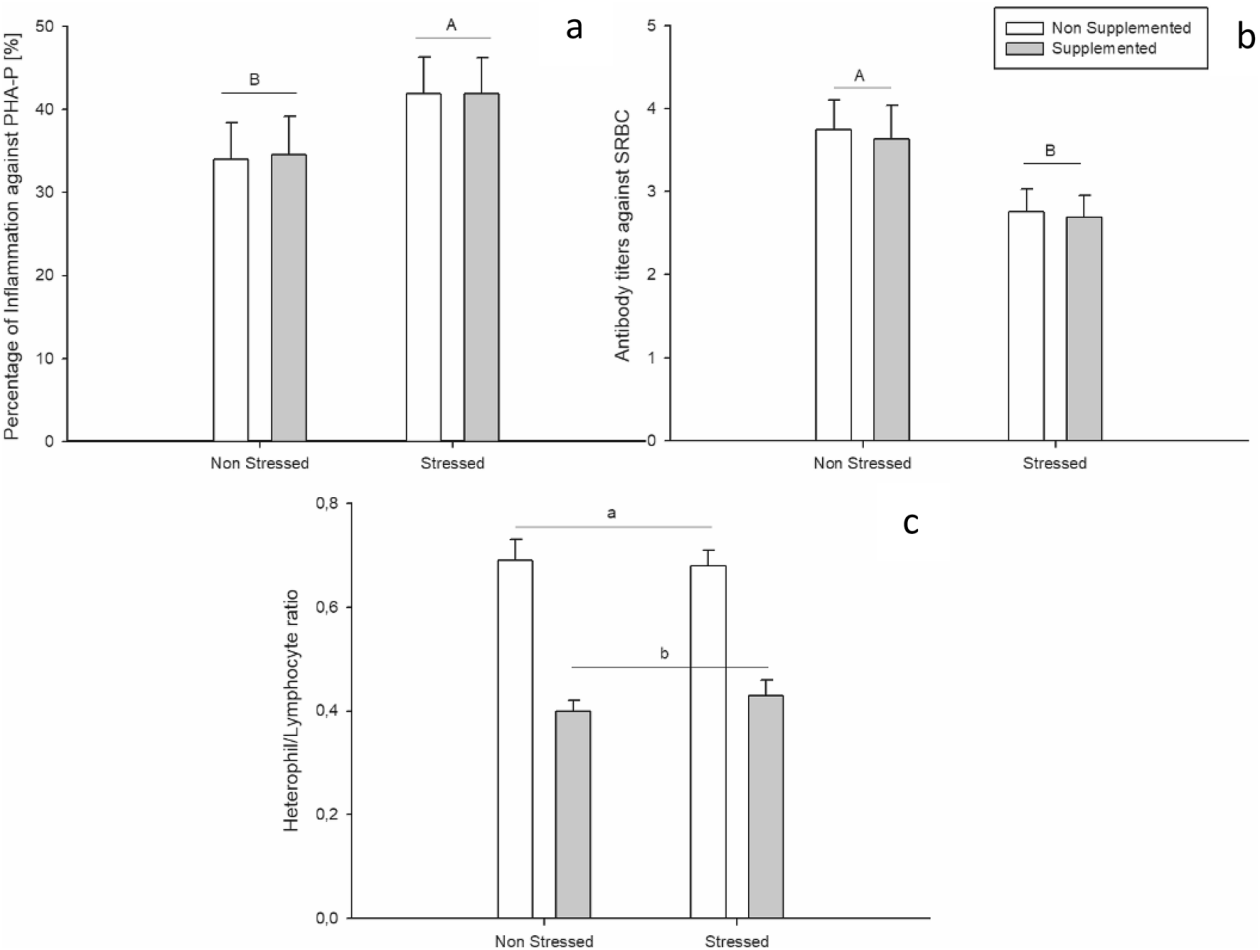
Effects of thymol dietary supplementation and the environmental challenges in the parental generation on the percentage of inflammation against PHA-P (**a**), titters against SRBC (**b**) and heterophil/lymphocyte ratio (**c**) in their undisturbed offspring. Number of chicks per treatment ranged from 15 to 25. Bars represent the mean value and lines represent standard error. In the parental generation: Dietary supplementation started at 103 days of age and lasted until the end of the study (130 days of age). Quail received a thymol dose equivalent to 80 mg/quail per day. At 115 days of age, quail were inoculated with Salmonella Enteritidis (SE) by an intramuscular injection. Chronic heat stress was achieved increasing room temperature from 24 to 34 °C during 9 consecutive days (121–129 days of age). Considering that no main effects of SE inoculation were detected, both SE and non-SE animals were pooled within the figure. ^A–B/a–b^Represents statistical differences (P < 0.05) between groups.

The number of chicks corresponding to each one of the parental groups were similar between the treatments (F < 1.17, P-value > 0.28), with an average number of 2.52 ± 1.23 chicks per parental pair. The analysis of the inflammatory response against PHA-P and the titers against SRBC on undisturbed chicks showed no effects of exposing their parents to a thymol supplementation and no interactive effects between their supplementation and exposure to inoculation and/or CHS challenges. However, regardless of the other treatments, a main effect of exposing the parents to CHS was found on the chicks’ inflammatory response (F = 5.97; P = 0.01; Fig. 3a) and on titers against SRBC (F = 11.26; P = 0.01; Fig. 3b). The chicks whose parents were exposed to CHS respectively showed a higher inflammatory response and lower titers than the chicks whose parents were not exposed to CHS.

For the H/L ratio (Fig. 3c), the statistical analysis showed a main effect of dietary supplementation (F = 65.00; P < 0.001) and no interactive effects with the other factors. Those chicks whose parents were supplemented with thymol showed lower values of the H/L ratio than those chicks whose parents were not supplemented.

## Discussion

This study was aimed at assessing whether feed supplementation with thymol can positively modulate the immune response of adult quails when exposed to simultaneous environmental challenges (CHS exposure and inoculation with inactivated *Salmonella* Enteritidis). In addition, transgenerational effects of the experienced situations were evaluated on immune representative variables on their eggs and offspring.

Each of the selected environmental challenges has previously been described to affect the physiology of birds, having major impacts on growth, development, metabolism and behavior^14,16–18,29–32^. Considering the *Salmonella* inoculum as an immune challenge and the exposure to high temperatures as a chronic stressor, reported negative consequences on the physiology of birds, in particular in poultry species, such as diminishing the feed intake, and therefore, affecting the weight gain and the feed conversion^12,14,18,19^. As for thymol, beneficial effects have been reported when this natural compound was included in the diet of poultry species, improving not only performance variables such as weight gain and the stability of fatty acids, but also reducing the fear-related responses without affecting the locomotion activity^27–30^. In our study, the assessment of immune variables in the parental generation showed baseline gender differences for the titers against SRBC and the H/L ratio. Females showed a stronger and more robust response against the antigen and, despite the inoculum, higher values of H/L ratio than males. However, and unlike females, when males were inoculated with *S.* Enteritidis showed higher H/L ratio than non-inoculated males. Our results, together with the previously reported differences between sexes^6,7^, indicate that males would be more susceptible to potentially harmful environmental pathogens or antigens, evidenced by an elevated H/L ratio which is indicative of increased stress response mediators concentration (the higher the values of the indicator, the higher the plasmatic mediators concentration^46,47^). Our results agree with previous reports informing that the exposure to CHS impairs the inflammatory response against PHA-P and the H/L ratio in adult Japanese quail^11,24,33^. Regarding supplementation under thermoneutral (control) conditions, previous reports have demonstrated an enhanced inflammatory and humoral response when poultry species were supplemented with thymol or a combination of thymol and its isomer, carvacrol^34,35^. Nevertheless, we found no immune differences between supplemented and non-supplemented control adults. It is important to mention that inflammatory response against PHA-P is a reliable index of the birds pro-inflammatory potential^48^, while H/L ratio is a classic indicator of circulating stress mediators. This fact implies that the immune responses evaluated under control conditions are not being altered by the thymol dietary supplementation, neither positively nor negatively.

However, when birds were exposed to high environmental temperatures (CHS groups), we observed the suggested thymol immunomodulatory and immuno-stimulatory effects previously reported^34,35^. The supplementation with thymol induced in the CHS treated parents a partial improvement on their inflammatory response and prevented an increment in the H/L ratio compared to their non-supplemented counterparts. Thus, thymol supplement seems to be beneficial when birds are exposed to heat stress conditions by preventing the suppression of the inflammatory responses, and therefore partially maintaining the capability of neutralizing and eliminating potentially harmful microorganism^2,35^. In this regard, it has been demonstrated that in birds both heat stress and supplementation with phytogenic compounds may affect reactive oxygen species (ROS)-dependent cell signaling pathways and transcription factors^49–51^. When applied together certain combinations of heat stress levels (acute or chronic, chronic-cyclic or chronic-constant) and supplement (dose and duration of supplementation protocol), could increase and decrease the expression of liver transcription factors NF-κB and Nrf2, respectively. As observed in animals supplemented with thymol and exposed to heat stress herein modulation of the expression of these factors may lead to prevent the suppression of the inflammatory response (but not necessarily affect inflammatory signaling). Hence, a better understanding of the impact of different levels of heat stress and supplementation on cell signaling warrants attention, since effects and mechanisms when both factors are applied together seem to be different to those imparted by each factor separately^49,52,53^. Regarding H/L ratio, previous studies informed that thymol can also impact on the central nervous system by interacting with the GABA receptor^36,54,55^. Under high environmental temperatures, these mechanisms could be enhanced to help birds reducing anxiety levels and to better cope with the challenge. Therefore, although it was not demonstrated in this study, the results are consistent with no changes in the plasmatic concentrations of stress mediators that could explain why the distribution of the leukocyte populations was not altered.

In general, the physiological responses associated with the activation of the INE interplay always demand energy and resources, in order to cope with the influence of a stressor and, thereafter, the reestablishing of baseline conditions. Under the exposure of a potential stressor, whatever its nature is, the activation of the INE interplay triggers a series of physiological and behavioral changes in order to deal with its influence^9,56^. Consequently, the organism must differentially allocate resources, both metabolic and energetic, prioritizing in some cases survival, in other cases, reproduction, evidencing some trade-offs^9,57,58^. In our study, the inoculum of *Salmonella* affected the H/L ratio of males, having those males exposed to the immune challenge higher values than those which were not exposed to it. However, the simultaneous exposure to the immune challenge and the CHS protocol did not further affect the immune responses of the adults belonging to the parental generation. Even though the adults, especially those under the exposure of high environmental conditions, had an impaired immune response, the environmental factors did not affect, either positively or negatively, egg laying, as there were no significant differences between the number of eggs collected during the last 5 days of the CHS protocol and the day after it ended. According to our results, it seems that the parental generation would be prioritizing energy and resources for the next generation even if this means a detriment on their current potential of response.

As mentioned before, eggs gather not only the essential components for the development of the embryo but also antimicrobial proteins, immunoglobulins, and even components from the maternal diet^40,59^. In our study, the parental generation that bred under thermoneutral conditions laid eggs with similar antibacterial activity regardless whether the birds were or not supplemented. The agglutinant activity of the eggs was increased when the parental generation was supplemented with thymol, independently from the parents being submitted to a CHS condition or not. The antimicrobial activity is part of the innate branch of the immune system that provides nonspecific protection against potentially pathogens^45^. On the other hand, the agglutinant activity analyzed is part of the acquired branch of the immune system that in this work was assessed by the production of specific antibodies against *Salmonella* Enteritidis^60^. Results suggest that supplementing thymol to the parental generation improves preferably a specific humoral immune response against *Salmonella,* not affecting/modulating the unspecific response of the antibacterial activity general response against other potentially harmful pathogens in the eggs.

Antibacterial activity, without thymol supplementation, and agglutinating activity, independently of the thymol supplementation, were both increased by the exposure of the parental generation to the CHS protocol. It is important to consider that the exposure to high environmental temperatures impaired the immune response of the parental generation (decreasing their inflammatory response and increasing the H/L ratio). Therefore, our results agree with previous reports suggesting that when reproductively active adults are under challenging environmental conditions, they divert energy and resources to protect their progeny rather than to mount an optimal immune response^61–63^. However, when the parental generation was supplemented, thymol positively modulated the inflammatory response against PHA-P and prevented the increase of the H/L ratio. Similar results were observed in the eggs from non-stressed mothers. Thus, it is suggested that thymol supplementation helped parental generation to better cope with the heat stress challenging conditions and therefore, the “transgenerational efforts” could be fully directed to increase the acquired immune response against specific pathogens.

In avian species, all the maternal components are transferred from mothers to their chicks through the egg^2^. Particularly, the immune components last for the first 2–3 weeks of age, providing protection against potential threats to which the parental generation was exposed^64^. During this time frame, Rubolini et al. (2005) showed that elevated concentrations of corticosterone in eggs suppressed the inflammatory response against PHA but had no effect on the humoral response against Newcastle disease^65^. Chicks gain independence from these maternal components as they become capable of mounting their own immune responses. In our study, at 32 days of age, once the offspring is immunologically completely independent, those chicks whose parents were exposed to high environmental temperatures for 9 consecutive days showed an increased inflammatory response against PHA-P and lower production of antibodies against SRBC. These findings are consistent with a parental generation “programming” of their chicks to mount an innate immune response rather than an acquired one, developing a quick and unspecific response which would demand less energy and resources to deal with potential pathogens^2^.

As expected, the H/L ratio observed in the chicks remained at basal levels^24^, since they were not exposed to any environmental challenge. However, although they were not intentionally stressed, some stress responses would be expected throughout their development. For example, non-specific systemic stress responses are anticipated for routine maintenance chores (e.g., during daily manual feed replenishment, pen box cleaning, etc.), during bird capture, crating, transport and handling for the purposes of hatching, leg and wing banding, and housing, and also as a result of placement into novel groups (e.g., hatching baskets and brooders) with the attendant modification of social structures^7,65–67^. Interestingly, chicks whose parents were supplemented with thymol showed lower H/L values than those from non-supplemented parents. This reduced H/L ratio (consistent with lowered concentrations of stress response mediators) suggest that supplementation of the parents’ diet with thymol may impact on their offspring, probably helping them to better deal with the unavoidable stressors during development. Based on the observed results some questions arise: would these chicks be able to deal better with other future challenging conditions? Moreover, if the offspring were to be exposed to the same environmental conditions than the parental birds, would the chicks show transgenerational effects that allow them to better cope with those same challenges? The answers of these questions might be found within the environmental match/miss-match hypothesis. Considering that the transgenerational or maternal effects are signaling and programming the offspring to develop their daily activities in a similar environment where the adult generation was raised, potential similarities and differences between the pre-natal and post-natal environment might be influencing the offspring^38,68^. Previous research in this field has found that an environmental mis-match, in terms of increased levels of CORT and different access to food, between the environment where the parental generation was raised and the environment where the offspring was raised impacted on growth, physiology and development of the offspring^38,68^. However, further trials are needed in this line to explore and better understand the phenomenon under study and their potential implications considering the environmental factors used in our study.

In conclusion, environmental conditions can modulate immune responses in the parental generation and may also have transgenerational effects on their offspring. Dietary supplementation with thymol partially and positively modulated the inflammatory response against PHA-P and avoided H/L ratio alteration in the parental generation exposed to high environmental temperatures, suggesting these adults dealt better with the challenge. The fact that adults coped better with the stressor influenced eggs’ antibacterial activity, showing similar values to those eggs laid by females under thermoneutral conditions. Regardless breeding conditions, thymol increased the agglutinating activity against *Salmonella* therefore giving protection to a specific pathogen. The supplementation of adults with thymol also had transgenerational effects on their offspring, having lower values of the H/L ratio. This suggest that chicks are more capable to deal with potential stressful situations associated with conventional breeding conditions.

## Materials and methods

### Animals and husbandry

Adult (parental generation) and juvenile (offspring) Japanese quail (*Coturnix coturnix*) were used in this study as an avian animal model because of their small size, their short life cycle and their low cost of maintenance^69^. Quail were raised according to standard laboratory protocols^44,70^. Animal care was provided in adherence with Institutional Animal Care and Use Committee guidelines. The experiment procedures were approved by the ethical committee at Instituto de Investigaciones Biológicas y Tecnológicas (IIByT) in compliance with the legislation regarding the use of animals for experimental and other scientific purposes (Acta 27, 17/11/2016).

### Parental generation

A total of 83 female:male Japanese quail pairs of 100 days of age were housed in enriched cages measuring 45 × 20 × 25 cm (long × wide × high), with the 50% of the floor covered by corrugated cardboard and a plastic ball^7,71^. At that age, all females were laying eggs and picked egg production (between 5 and 7 eggs per week). Food and water were provided ad libitum throughout the experiment. Birds were maintained on a daily cycle of 14 h light (300–320 lx) and 10 h dark during the whole study, lights switching on at 06:00 hs am. The room temperature was kept at 25 °C (comfort temperature). For habituation to the breeding cages, the birds were located in the experimental facilities 3 days (100–102 DA) before experimental manipulations started.

**Figure 4:**
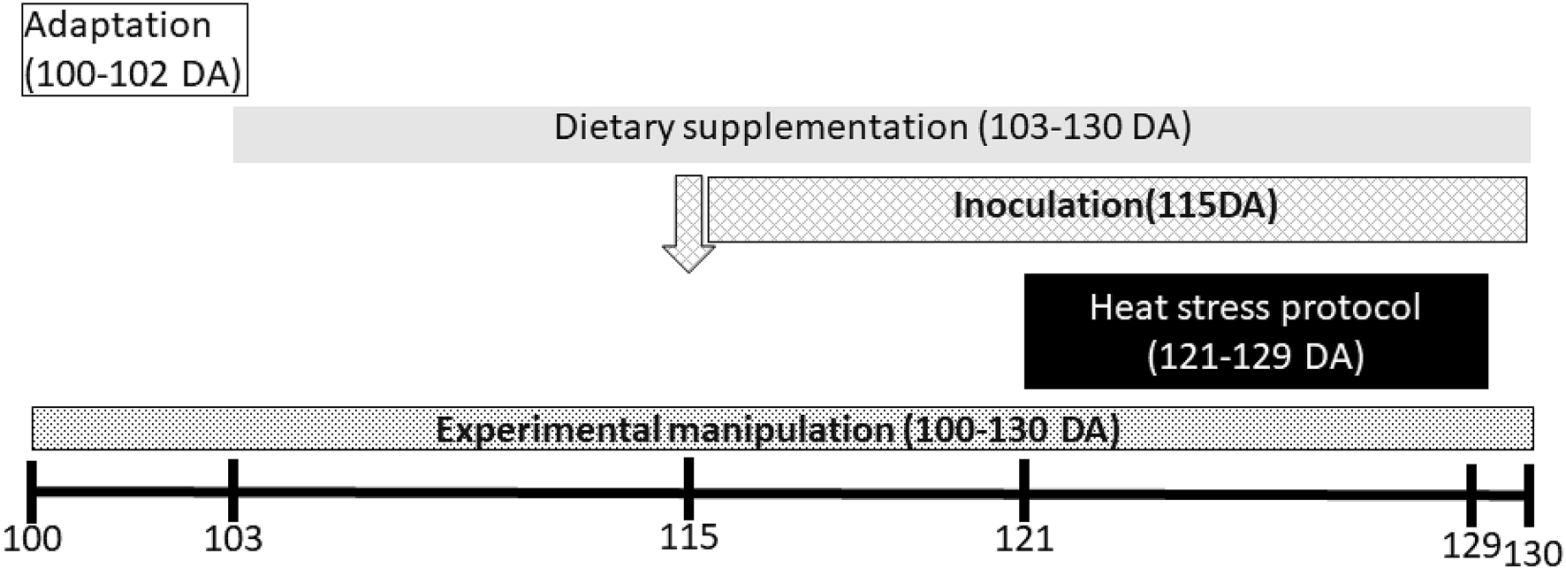
Timeline scheme. Dietary supplementation began at 103 days of age and continued until after blood samples were taken (130 days of age). Quail received a thymol dose equivalent to 80 mg/quail per day. At 115 days of age, quail were inoculated with Salmonella Enteritidis by intramuscular injection. Heat stress protocol was applied during 9 consecutive days (119–127 days of age) and achieved by increasing room temperature from 24 to 34 °C during daylight hours.

A 3 × 2 factorial experimental design (Fig. 4) was established with Dietary Supplementation (Non Supplemented vs. Supplemented), Inoculum (Non Inoculated vs. Inoculated) and CHS (Non Stressed vs. Stressed) as factors. Thus, 8 experimental groups were established resulting from the combination of treatments.

Dietary supplementation was provided following Nazar et al. (2018) protocol^24^. Briefly, thymol was commercially obtained from Sigma-Aldrich (SAFC, ≥ 99%; FCC, Saint Louis, MO, USA). Two g of thymol per kg of feed (equivalent to a dose of ≈ 80 mg of thymol/day per bird) was provided to supplemented quail diet. Thymol was prepared in a 2.4% w/v ethanolic solution and uniformly sprayed on fresh feed^31^. The feed was weighted and provided every 3 days in a ratio of ≈ 40 g/quail per day, according to the dietary treatment. The supplementation started at 103 days of age (13 days prior to the administration of the inoculum and 18 days prior to the beginning of the CHS protocol, in order to reach the physiological plateau or stationary state of thymol in the organism and ensuring thymol transference to the eff yolk^44,72^). The supplementation continued until blood samples were obtained (130 days of age, see below).

Inoculum was administered at 115 days of age, 15 days before the blood samples were taken. Three hundred µl of a suspension of formalin-inactivated *Salmonella* ser. Enteritidis (10^8^ bacteria/quail) were intramuscularly inoculated in the male and female right breast, according to the previous treatment assignation^60^. The adults that were not inoculated against *Salmonella* Enteritidis were handled as their counterparts, but were not exposed to that immune challenge.

The CHS protocol was performed according to Nazar et al. (2018)^24^. Briefly, room temperature was increased from 25 to 34 °C during a 2-h period starting at 8:30 h and maintained at 34 °C until 16:30 h. At 16:30 h temperature was gradually lowered returning to 24 °C approximately by 19:30 h. The CHS protocol was developed during 9 continuous days (121–129 days of age).

### Offspring

Eggs from the parental generation were collected during days 5, 6, 7, 8 and 9 of the CHS protocol (125–129 days of age) and on day 130 of age¸ when also blood samples were taken; thus sampling included six continued days. It is assumed that the eggs collected on last day also gathered all the information experienced by the females during the previous day (day 9 of CHS protocol). All eggs were properly labeled to identify the offspring associated to each of the 8 treatments. There were no significant differences between the amount of eggs laid by females in each treatment (Generalized Linear Model, Poisson distribution, F < 2.04, P-values > 0.15). A total of 5 egg per treatment (5 × 8 = 40 egg total) collected on the ninth day of the CHS protocol were used to analyze the effects of the treatments on the egg immune variables (see below). The other eggs were artificially incubated according to standard laboratory procedures^73^. At 10 a.m. of hatching day, chicks were leg-banded to track their parental treatments. Quail chicks were randomly housed in three mixed-parental treatment rearing pen boxes measuring 90 × 90 × 60 cm (length × width × height). Each box had 1 feeder of 90 cm and 16 automatic nipple drinkers. A wire-mesh floor (1 cm grid) was raised 5 cm to allow the passage of excreta and a lid prevented the birds from escaping. Brooding temperature was 37 °C during the first week of life, with a decline of 3 °C per week until 25 °C was reached. For the first 4 weeks of life, quail starter diet (24% CP; 2900 kcal ME/kg) and water were provided ad libitum. Quail were subjected to a daily cycle of 14 h light (300–320 lx) and 10 h dark throughout the study. It is important to mention that juvenile chicks were raised undisturbed and were not exposed to any of the environmental challenges that their parental generation were exposed to. A total of 21 Non-supplemented/Non-Inoculated/Non-Stressed, 17 Non-supplemented/Inoculated/Non-Stressed, 15 Supplemented/Non-Inoculated/Non-Stressed, 21 Supplemented/ Inoculated/Non-Stressed, 25 Non-supplemented/Non-Inoculated/Stressed, 18 Non-supplemented/Inoculated/Stressed, 22 Supplemented/Non-Inoculated/Stressed, 20 Supplemented/Inoculated/Stressed chickens were evaluated. There were no differences between the number of chicks corresponding to each one of the treatments (Generalized Linear Model, Poisson distribution, F < 1.17, P-value > 0.28; the average number of chicks was 2.52 ± 1.23)|.

### Blood sampling

Blood samples were taken using a 1 ml sterile syringe previously treated with ethylenediaminetetraacetic acid (EDTA), by collecting blood from the jugular vein of each quail. One blood drop was used for smears preparation, and the remaining was centrifuged at 2000*g* during 15 min in order to obtain plasma for later determinations of titers against SRBC previously induced (see below). In adults, blood sampling was performed at 130 days of age (the day after the CHS protocol was ended) whereas in the offspring, the blood sampling was performed at 32 days of age.

### Immune variables evaluated in both, the parental generation and their offspring

The inflammatory response against PHA-P (Sigma Chemical, St Louis, MO, USA; pH: 7.1) was assessed as described by Nazar et al. (2018)^24^. Briefly, 24 h before blood sampling, the right wing web was measured with a digital caliper, and then 0.05 ml of a 1 mg/ml PHA-P solution in buffer phosphate saline was injected. Twenty-four hours later and immediately after blood sampling, the corresponding wing web was again measured to determine the percentage of inflammation induced.

Titers against SRBC (HEMO-G; Rafaela, Santa Fe, Argentina) were analyzed by microtiter agglutination assay using a round bottom microagglutination plate. A week before the blood sampling procedure, 300 µl of a 10% solution of SRBC was intraperitoneally injected. The microagglutination assay was performed according to Nazar et al. (2018)^24^.

The blood smears previously prepared were stained with May-Grundwal Giemsa. White blood cells populations (A total of 100 cells), such as lymphocytes, heterophils, eosinophils, basophils and monocytes were counted using a white light optical microscope at 400 × magnification. H/L ratio was then calculated for each quail.

### Immune variables evaluated in eggs

Antibacterial activity was measured using the method described by Shawkey et al. (2008), slightly modified^74^. An *Escherichia coli* strain was incubated overnight at 37 °C at 160 rpm in a sterile medium containing 50 ml of distilled water, 0.5 g of triptone, 0.25 g of yeast extract and 0.5 g of NaCl. Then, this inoculum was used to prepare a solution with a final absorbance of 0.7 at 600 nm. This solution was used to perform the assay in flat-bottomed 96 wells plates. Briefly, in each well 80 µl of the bacterial solution were mixed with 20 µl of white egg from the 5 eggs per treatment (40 eggs total). The plate was incubated overnight at 37 °C and then the optical density was measured at 600 nm using a Multiskan Spectrum v1.2 microplate reader. The antibacterial activity of the samples was compared to the blank (80 µl of physiological solution and 20 µl of white egg) and negative control (80 µl with culture suspension and 20 µl of physiological solution). It is important to consider that the lower the values of the variable, the higher the inhibition of the bacterial growth^45^.

To measure the immunogenicity against *Salmonella* Enteritidis; egg yolks were homogenized 1:5 with distilled water. Thereafter titers against the microorganism were determined by microtiter plate agglutination using U-bottomed 96-well plates. Briefly, 50 µl of twofold serial dilutions of the egg yolk samples were mixed thoroughly with 50 µl of *Salmonella* Enteritidis inactivated antigen (*circa* 10^8^ cells/ml). Plates were incubated at 37 °C for 18 h, and the agglutinant titer was determined as the highest dilution of the egg yolk for which evident agglutination was observed.

### Statistical analysis

All variables were analyzed using Generalized Linear Mixed Models, with dietary supplementation, the inoculum and CHS included as fixed effects. The analysis of the variables evaluated in the parental generation and eggs also included the identity of cages as a random effect and the analysis of offspring variables included the pen boxes identity as random effect. Sex was also included in the parental generation as a fixed effect due to the expected differences between males and females. Each variable was analyzed according to their distribution of data. P-value of < 0.05 was considered to represent significant differences. LSD Fisher test was used for post hoc comparisons. All statistical analyses were performed with an ‘R’ (The R Foundation for Statistical Computing) user-friendly interface implemented in InfoStat^64^.
